# Understanding the limits of binary diffusion for enhanced clay barrier design

**DOI:** 10.1093/pnasnexus/pgae366

**Published:** 2024-08-23

**Authors:** Jooyoung Im, J Carlos Santamarina

**Affiliations:** Energy Resources & Petroleum Engineering, King Abdullah University of Science and Technology (KAUST), Thuwal 23955, Saudi Arabia; School of Civil and Environmental Engineering, Georgia Institute of Technology, Atlanta, GA 30332, USA

**Keywords:** diffusion, waste containment, coupled processes

## Abstract

Waste containment and isolation strategies often utilize bentonite as a buffer material due to its swelling capacity, sealing efficiency, low permeability, and limited diffusive transport. However, previous experimental studies of ionic diffusion through bentonite have shown discrepancies with binary diffusion assumptions. Meticulous experiments and complementary analyses reveal that the migration of preexisting ions in the medium enables the differential flux of diffusing anions and cations, while maintaining local electroneutrality in all cases. The separation between the cationic and anionic fronts is electrically tied to the motion of the preexisting ions and reflects the interplay between valence, concentration, and self-diffusion coefficients of the ions involved. Imposing binary diffusion conditions forces the faster anions to diffuse at the same rate as cations. Therefore, effective barriers to mitigate both cation and anion transport should have low surface charge and low excess salts to minimize the preexisting ionic concentration.

**Significance Statement**

High-specific-surface area clays are widely used in the containment of hazardous materials. Previous studies have explored various modifications to enhance their sealing capacity, for example, by modifying the surface charges; yet, these modifications often aggravate the diffusive transport of contaminants and may inadvertently lead to increased release rates. The detailed understanding of electrostatic interactions among diffusing ions and preexisting ions in clay barriers remains lacking. Results highlight the extent of electrostatic interaction among all coexisting ionic species and their pronounced implications on diffusive transport. The comprehensive understanding of electrostatically coupled diffusive transport in clays creates new opportunities for the design of more efficient and sustainable waste management solutions.

## Introduction

The global surge in both human population and consumption per capita (1) has accelerated the generation of hazardous and nonhazardous waste (2). Hazardous waste disposal requires safe containment to avert its release into the environment (3). Bentonite is a common component of engineered barriers for near surface landfills and deep repositories (4–7) because of its low hydraulic conductivity, swelling potential, high sealing capacity, and effective sorption of cations (8–11). However, it has a lower efficacy against anionic species like radioisotopes of iodine or pertechnetate (12, 13). Research and development efforts devoted to modifying bentonite to enhance its performance against anionic species have involved altering the surface charge of bentonite to a more positive state (14–16). However, such modifications diminish its effectiveness against cation transport (17).

Diffusion governs the migration of ionic species in the absence of advection or convection. When cations and anions diffuse at differing rates, a local electric field arises from charge separation. This field induces electrostatic-driven migration causing the “slower” ion to speed up and the “faster” ion to slow down in binary diffusion. As a result, both ions diffuse at similar rates maintaining local electroneutrality (18). Some experimental results confirm binary diffusion through bentonite specimens (19); however, other studies show conflicting results (20, 21). Apparently, unmeasured “preexisting” ions within the clay barrier become involved. Previous studies using molecular dynamics simulations have shown the effect of coexisting species on their self-diffusion (22); however, detailed experimental studies are still lacking.

The purpose of this study is to gain a comprehensive understanding of ionic diffusion in bentonite. Ultimately, the goal is to engineer barriers to effectively mitigate the transport of both cationic and anionic species.

## Materials and methods

### Materials

The primary clay used is a bentonite composed of mainly montmorillonite. The elemental mass-fraction composition is: 77% SiO_2_, 13% Al_2_O_3_, 3.8% Fe_2_O_3_, 1.4% MgO, 1.5% Na_2_O, 1.3% CaO, 0.5% K_2_O, and 0.2% SO_3_. Its hydrated specific surface area is 593 m^2^/g (crystal violet colorimetry (23)), and its liquid limit (LL) is 390 (standard fall cone test (24)). The bentonite is prewashed in deionized water and centrifuged two times to remove excess salts. Na^+^ (42%) and Ca^2+^ (48%) are the main preexisting counterions to balance the available negative surface charge, together with some K^+^ (10%).

We conduct one complementary test using dry-processed kaolin washed with deionized water to remove excess salts (test 9: composition: 67% SiO_2_, 29% Al_2_O_3_, 1.5% TiO_2_, 0.9% Fe_2_O_3_, 0.2% K_2_O, 0.07% CaO, and 0.06% SO_3_; hydrated specific surface area: 34 m^2^/g; LL: 48). The surface charge is balanced by potassium K^+^, i.e. the preexisting ions in the washed kaolinite.

The ions selected for diffusion tests are K^+^, Cs^+^, Sr^2+^, Cl^−^, and ${\mathrm{SO}}_{4}^{2-}$. These ions are introduced as high salinity aqueous solutions prepared with deionized water to facilitate the detection of electrochemically coupled diffusive transport. Preexisting ions compensate the mineral surface charge and form precipitated excess salts in unwashed dry clays (25).

### Methods

We conduct diffusion experiments using two contiguous cylindrical cells (*d* = 50 mm, *h* = 20 mm). Tests are designed to focus on differential diffusion rates. Therefore, we precisely control the volumetric ionic concentration in both cylindrical cells while limiting any unwanted effects from changes in fabric, density, porosity, or water content.

Clay pastes are prepared by thoroughly mixing dry clay powder with the selected solution at 70% water content by weight. This results in an average dry density of 1.0 ± 0.08 g/cm^3^, an average degree of saturation of 97%, and an average porosity of 0.62 for the bentonite samples. Kaolin test (test 9) has a dry density of 0.99 g/cm^3^, degree of saturation of 99%, and porosity of 0.62. The 70% water content is chosen to ensure that the bentonite paste prepared with high salinity solutions (up to 1 M) does not exceed the LL, while still allowing sufficient homogeneous mixing of the bentonite with deionized water (tests 1–4). The selected test conditions result in similar mineral “dry” densities across all specimens, help preserve the clay fabric during the test, and prevent fluid leakage.

The clay powder and target solution are thoroughly mixed within a sealed bag to avoid moisture loss. The mixture is then used to fill cylinders, and pressed to reach the target density (the resulting specimens can be deformed with hand pressure). Each cylinder is individually sealed to prevent evaporation and left to homogenize at room temperature for 72 h. After homogenization, two distinct cylinders are aligned and brought together; the tested clays are water wet and displace the nonwetting air upon approach; a light load presses specimens together to ensure an adequate contact. Data repeatability and the evolving continuity of concentration profiles suggest that proper contact prevailed in all tests. Diffusion is allowed to progress at 20 °C for 8 to 24 h, ensuring that the diffusing ions remain within ∼15 mm from the interface and away from the end boundaries.

After the allocated diffusion time, we sample specimens by successively “shaving” 0.5 mm thick layers normal to the direction of diffusion, and analyze all samples using Wavelength Dispersive X-Ray Fluorescence (WD-XRF Bruker S8 Tiger Series 2). We measure the diffusing ions as well as preexisting ions, including Na^+^, Ca^2+^, K^+^, and ${\mathrm{SO}}_{4}^{2-}$.

Finally, the diffusion coefficient $D_{i}$ (m^2^/s) is obtained by fitting the measured concentration profiles using the solution to the 1D diffusion equation:


(1) $$\frac{c_{x}}{c_{0}}=0.5\mathrm{erfc}\left[\frac{x}{2\sqrt{D_{i}t}}\right]$$

in terms of the concentration $c_{x}$ (moles/m^3^) at a distance *x* (mm) from the interface, the initial concentration $c_{0}$ (moles/m^3^), and the test duration *t* (s).

## Tested systems—results

We designed tests to examine various diffusion hypotheses. Table 1 summarizes the tested two-cylinder configurations. Tests 1 to 4 introduce the target salt to the left half-cylinder to investigate the impact of ion valence and mobility on diffusion coefficients. Tests 5 to 8 involve dosing both sides with target salts at concentrations designed to allow the diffusion of two ions only: same K^+^ concentration on both sides to explore the diffusion of Cl^−^ against ${\mathrm{SO}}_{4}^{2-}$ (test 5), and same Cl^−^ concentrations on both sides to study the counter diffusion of various cations (tests 6–8). Finally, tests 9–15 control the concentration of preexisting ions to assess their effect on the diffusion of other species. Test 9 utilizes washed kaolin instead of bentonite to lower the concentration of preexisting ions, i.e. surface charge density multiplied by the specific surface area. Tests 10 to 14 introduce sodium salts to both sides to elevate the concentration of preexisting ions.

**Table 1.** Overall testing conditions and inferred diffusion coefficients.

Test #	Test conditions				Preexisting ionic strength (moles/m^3^)	Inferred diffusion coefficient *D_i_* × 10^−11^ (m^2^/s)					α = *D*_Fast_/*D*_Slow_.
	Clay	Avg. water content (deg. of sat.) (%)	Left (molality)	Right (molality)		*K* ^+^	Sr^2+^	Cs^+^	Cl^−^	SO_4_^2−^	
1	Washed Na-bentonite	68.1 (98.7)	KCl (1 M)	DI	600 (no excess salts Na^+^ is the prevalent counterion)	16.0	—	—	16.0	—	1.0
2		68.7 (97.7)	K_2_SO_4_ (0.5 M)	DI		16.0	—	—	—	8.0	2.0
3		66.0 (97.7)	SrCl_2_ (0.5 M)	DI		—	7.5	—	14.5	—	1.9
4		65.3 (97.3)	CsCl (1 M)	DI		—	—	4.0	9.0	—	2.3
5		65.6 (97.0)	KCl (1 M)	K_2_SO_4_ (0.5 M)		—	—	—	34.0	16.5	2.1
6		67.2 (98.7)	KCl (1 M)	SrCl_2_ (0.5 M)		15.5	8.0	—	—	—	1.9
7		64.9 (98.4)	CsCl (1 M)	KCl (1 M)		15.0	—	8.0	—	—	1.9
8		64.9 (98.4)	CsCl (1 M)	SrCl_2_ (0.5 M)		—	14.0	7.0	—	—	2.0
9	K-Kaolin	62.0 (99.2)	CsCl (1 M)	DI	48	—	—	4.0	4.0	—	1.0
10	Washed Na-bentonite	64.2 (99.0)	CsCl (1 M), Na_2_SO_4_ (0.175 M)	Na_2_SO_4_ (0.175 M)	1,008	—	—	5.5	25.0	—	4.5
11		62.0 (99.8)	CsCl (1 M), Na_2_SO_4_ (0.25 M)	Na_2_SO_4_ (0.25 M)	1,281	—	—	5.5	45.0	—	8.2
12		64.9 (96.7)	Cs_2_SO_4_ (0.5 M), NaCl (0.25 M)	NaCl (0.25 M)	950	—	—	5.5	—	18.0	3.3
13		61.8 (99.6)	Cs_2_SO_4_ (0.5 M), NaCl (0.5 M)	NaCl (0.5 M)	1,247	—	—	5.0	—	25.0	5.0
14		59.8 (98.9)	CsCl (1 M), NaCl (0.5 M)	NaCl (0.5 M)	1,247	—	—	5.0	45.0	—	9.0

DI , paste prepared with deionized water.

**Fig. 1.**
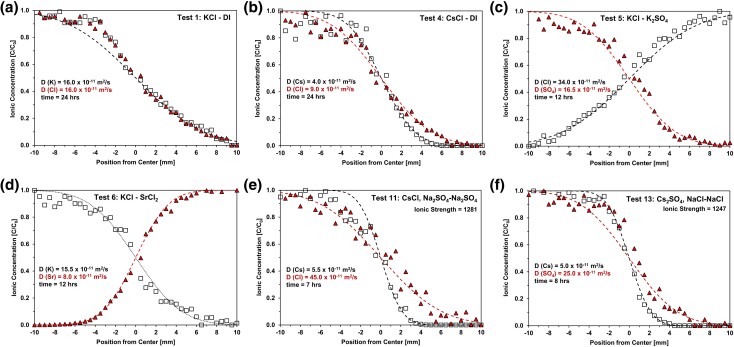
Concentration profiles after diffusion. (a and b) Tests 1 and 4: single salt. c) Test 5: anion into anion. d) Test 6: cation into cation. (e and f) Tests 11 and 14: preexisting sodium concentration. Inferred diffusion coefficients are identified in each case.

Figure 1 displays concentration profiles for 6 of the 14 tests reported herein (the supplementary figures contain the raw data and fitted diffusion curves for all tests): (a) the joint diffusion of K^+^ and Cl^−^, (b) differences in Cs^+^ and Cl^−^ co-diffusion, (c) the counter diffusion of mono and divalent anions Cl^−^ and ${\mathrm{SO}}_{4}^{2-}$, and (d) cations Cs^+^ and Sr^2+^, (e and f) differences in the diffusion of Cs^+^ together with either Cl^−^ or ${\mathrm{SO}}_{4}^{2-}$ in bentonite with a high concentration of preexisting ions.

Table 1 lists the diffusion coefficients inferred by fitting the measured concentration profiles using the solution to the 1D diffusion equation (Eq. 1). The results show that

- *Among various tests*: the same ionic species can exhibit markedly different diffusion coefficients depending on other coexisting ions and clay type; for example, within the tested conditions: K^+^ remains quite constant, Sr^+^ and Cs^+^ vary by a factor of 2, ${\mathrm{SO}}_{4}^{2-}$ by a factor of 3, and Cl^−^ varies by almost 10 times. Anions do not adsorb onto positively charged clay surfaces; hence, variations in anion diffusion coefficients must reflect electrostatic interactions with the diffusing cation and other preexisting ions. On the other hand, the diffusion coefficient for cations is also affected by the clay's cation exchange capacity.
- *For a given test*: diffusion coefficients differ for cations and anions (with the exception of tests 1 and 9—Table 1). The ratio between diffusion coefficients $\;\alpha =D_{\mathrm{Fast}}/D_{\mathrm{Slow}}$ exceeds *α* ≈ 2 in most cases. Differences in diffusion coefficient imply either a pronounced charge unbalance under binary diffusion conditions or the migration of preexisting ions to preserve local electroneutrality, as discussed next.

### Preexisting ions

**Fig. 2.**
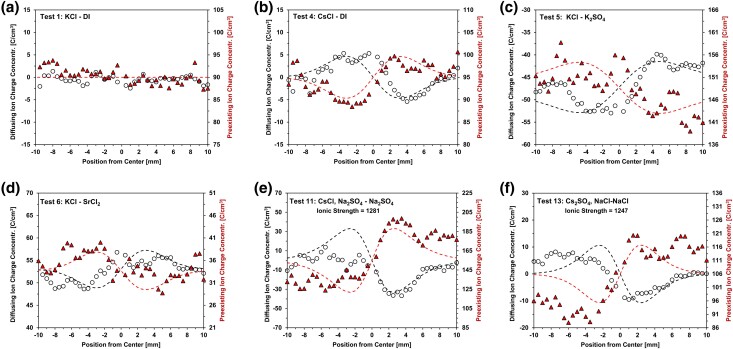
Charge distributions of diffusing and preexisting ions. (a and b) Tests 1 and 4: single salt. c) Test 5: anion into anion. d) Test 6: cation into cation. (e and f) Tests 11 and 14: preexisting sodium concentration.

Differences between the cationic and anionic concentrations of diffusing ions in Fig. 1 determine the local unbalanced charge concentration profiles plotted in Fig. 2 (the plotted data points are three-point averages to reduce the noise level associated with the Na^+^ resolution limit). These graphs also include the measured charge distribution for preexisting ions (see Supplementary material for the complete raw dataset). We fit the theoretical charge distribution caused by the measured diffusion coefficients and compute the complementary charge distribution to preserve local electroneutrality; these two trends are superimposed as dotted lines on Fig. 2. Indeed, local electroneutrality is preserved in all tests.

**Fig. 3.**
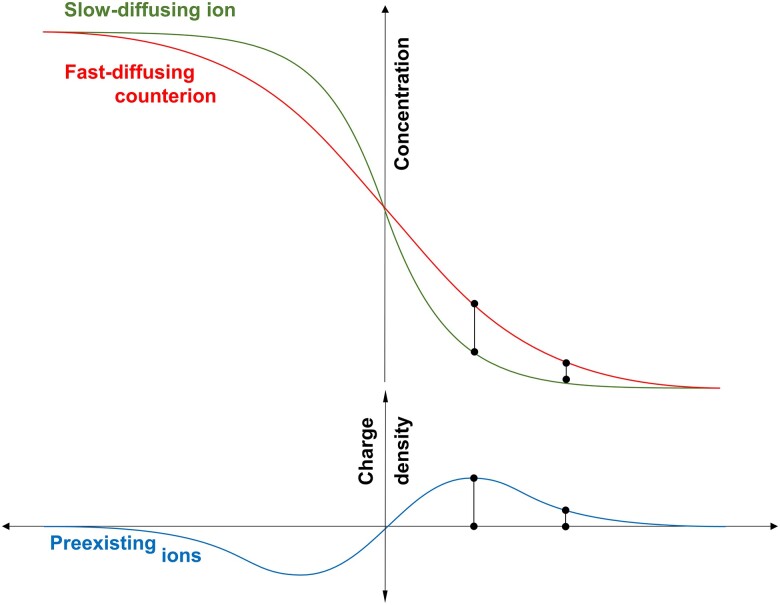
Nonbinary diffusion. The migration of preexisting ions enables the differential flux of diffusing anions and cations, while maintaining local electroneutrality throughout the medium.

Together, the charge distributions associated with diffusing ions and preexisting ions provide a comprehensive view of charge interaction throughout the specimen during diffusion. Remarkably, preexisting ions move to counterbalance charge discrepancies arising from the unequal diffusion rates of the diffusing ions. This is schematically summarized in Fig. 3.

Preexisting ions allow for significant discrepancy in the relative diffusion coefficients of cations and anions (see rows in Table 1), as well as marked variations in the diffusion coefficient of a specific ion under different conditions (see columns in Table 1). In the absence of preexisting ions, the fast-diffusing ions slow down and the lagging counterions are pulled ahead to satisfy local electroneutrality, thus both ions migrate with the same diffusion rate: $\alpha =D_{\mathrm{Fast}}/D_{\mathrm{Slow}}\approx \;1.0$. This was confirmed by creating quasi-binary diffusion conditions using a low specific surface clay washed with deionized water to remove excess salts (*k*-kaolin—test 9, Table 1).

**Fig. 4.**
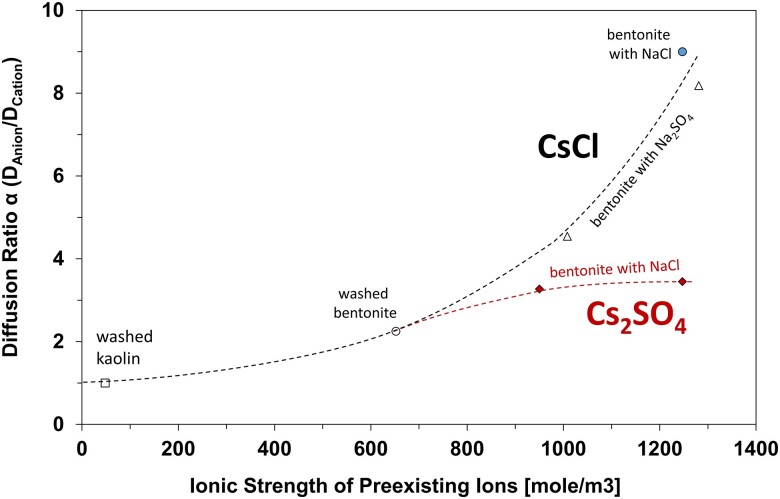
Diffusing ratio $\alpha =D_{\mathrm{Fast}}/D_{\mathrm{Slow}}$ as a function of the ionic strength of preexisting ions. Data for CsCl diffusion from tests 4, 9, 10, 11, 14, and 15. Data for Cs_2_SO_4_ diffusion from tests 12 and 13. Test details are summarized in Table 1.

Diffusion tests across specimens with increased preexisting ionic strength highlight the interplay between the diffusing ions and the preexisting ions (Table 1—added NaCl or Na_2_SO_4_ salts—Data in Supplementary material). Figure 4 illustrates that diffusion ratios $\alpha =D_{\mathrm{Cl}}/D_{\mathrm{Cs}}$ and $\alpha =D_{SO_{4}}/D_{\mathrm{Cs}}$ increase with the concentration of preexisting ions. We anticipate a maximum diffusion ratio $\alpha =D_{\mathrm{Fast}}/D_{\mathrm{Slow}}$ for any given salt, as observed for the diffusion of Cs_2_SO_4_ when the ionic strength of preexisting ions exceeds ∼1,000 moles/m^3^ (tests 12 and 13).

### From binary diffusion to diffusion in clays

The flux $J_{i}$ (mol/m^2^ s) of a specific species *i* is determined by both its Fickian diffusion $-D_{i}(dc_{i}/dx)$ and electrostatic-induced migration $z_{i}c_{i}(F/RT)(d\emptyset /dx)$. Assuming local electroneutrality and taking into consideration all *n*-species involved, the flux $J_{i}$ becomes (26)


(2) $$J_{i}=-D_{i}\left[\frac{dc_{i}}{dx}-z_{i}c_{i}\frac{\sum_{j}^{n}\;z_{j}D_{j}(dc_{j}/dx)}{\sum_{j}^{n}\;z_{j}^{2}D_{j}c_{j}}\right]$$

where each species has its own valence *z* (positive or negative), diffusion coefficient *D* (m^2^/s), and concentration *c* (mol/m^3^).

In the *absence of preexisting ions*, electroneutrality between a diffusing cation *A* and anion *B* requires $z_{A}c_{A}+z_{B}c_{B}=0$. Then, cations and anions migrate with the same effective diffusion coefficient $D^{*}$ in binary diffusion


(3) $$D_{\mathrm{anion}}^{*}\;=\;D_{\mathrm{cation}}^{*}\;=\;D_{A}\frac{z_{A}-z_{B}}{z_{A}(D_{A}/D_{B})-z_{B}}$$

In the *presence of preexisting ions*, diffusing ions can migrate at different rates as long as preexisting ions drift accordingly to satisfy electroneutrality (Eq. 2). Consider the diffusing cation *A* and anion *B*, and the preexisting ions *C* (e.g. the counterion cloud around charged clay surfaces). The displacement of preexisting ions will be electrostatic-induced and subject to self-diffusion. Then, the evolving local concentration gradients will be related as follows:


(4) $$\frac{dc_{C}}{dx}=z_{C}c_{C}\frac{z_{A}D_{A}dc_{A}/dx+z_{B}D_{B}dc_{B}/dx+z_{C}D_{C}dc_{C}/dx}{z_{A}^{2}D_{A}c_{A}+z_{B}^{2}D_{B}c_{B}+z_{C}^{2}D_{C}c_{C}}$$

Due to the opposite charges of cations and anions, Eq. 4 highlights that the concentration gradient of preexisting ions *C* increases with the growing difference in the diffusion coefficients of the diffusing ions *D_A_* and *D_B_*. Therefore, the development of a high concentration gradient (d*c_c_*/d*x*) in the preexisting ions *C* corresponds to a large separation among the diffusing ions *A* and *B*.

Experimental data confirm these observations. For example, consider tests 1–4 conducted in the same medium. Potassium has the highest diffusion coefficient among the tested cations and leads to the lowest value for the term $\;(z_{A}D_{A}[dc_{A}/dx]+z_{B}D_{B}[dc_{B}/dx])$. Consequently, the diffusion of KCl in test 1 results in the same diffusion coefficients for the cation K^+^ and anion Cl^−^ (*D* = 16.0×10^−11^ m^2^/s, *α* ≈ 1).

Equation 4 predicts proportionality between the evolving concentration gradient of preexisting ions *C* and their ionic strength. Indeed, experimental results for CsCl diffusion show preexisting ion migration in bentonite (test 9—ionic strength of preexisting ions: 650 moles/m^3^) but quasi-binary diffusion in kaolinite (test 9—ionic strength of preexisting ions: 48 moles/m^3^).

**Fig. 5.**
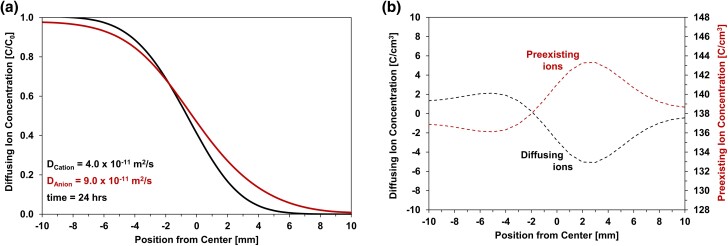
Differential cation and anion diffusion in the presence of preexisting ions—simulation (Eq. 2). a) Concentration profiles. b) Charge distributions.

Equation 4 constrains the conditions for binary diffusion, whereas Eq. 2 anticipates the separation between diffusing ions. Together, these equations capture the interplay between valence, concentration, and self-diffusion coefficients of ions involved in the overall diffusive flux. Simulated results based on the flux Eq. 2 are shown in Fig. 5; the diffusing anion and cation as well as the preexisting ions are monovalent with an initial concentration of 1 M, and the assumed diffusion coefficients are *D*_cation_ = 4.0 × 10^−11^ m^2^/s and *D*_anion_ = 9.0 × 10^−11^ m^2^/s (for the purposes of this simulation, *D*_preexisting_ >> *D*_anion_). The resulting charge distribution is asymmetric around the contact plane between the left and right specimen halves (Fig. 5b), thus, coupled ion fluxes deviate from Fickian diffusion in a strict sense.

### Implications

Anionic diffusion studies remain limited (27, 28). Yet, results above anticipate that anionic diffusive transport is electrically coupled to cationic diffusion and affected by preexisting ions and charges on mineral surfaces. More detailed analyses for diffusion in soft high-specific-surface media must consider other factors not addressed in this study such as preferential adsorption and fabric changes due to chemo-osmosis (29).

The experimental results and analyses advanced in this study highlight the effect of preexisting ions on the differential migration of diffusing anions and cations. For example, binary diffusion is achieved in test 9 using washed kaolin with a low concentration of preexisting ions; in this case, the chloride diffusion coefficient dropped to 4.0×10^−11^ m^2^/s (*α* ≈ 1) from the value of 9.0×10^−11^ m^2^/s (*α* ≈ 2.3) measured in the washed bentonite (test 4). In general, Eqs. 2 and 4 suggest opportunities for engineered barriers designed with controlled preexisting ions, both in terms of concentration and species. This suggests that effective barriers to mitigate both cation and anion transport should have not only low permeability, but also a low surface charge and low excess salts.

## Conclusions

Ions tend to migrate at their own species-specific diffusion rates. Differences in diffusion rates result in a local electrostatic field that affects all ions within the system. If only cations *A* and anions *B* are present, electrostatic interactions cause the diffusion of cations *A* and anions *B* to occur at the same rate (i.e. binary diffusion). However, the presence of preexisting ions *C* in the medium enables the differential diffusion of cations *A* and anions *B*, facilitated by the migration of the *C* ions. In both scenarios, local electroneutrality is preserved.

Detailed experiments conducted in this study reveal that local electroneutrality is maintained within clay barriers during diffusion. Electroneutrality combines the charges contributed by diffusing anions and cations, preexisting ions in the clay barrier (counter ions and excess salts), and the mineral surface charge.

The separation between the cationic and anionic fronts is electrically tied to the motion of the preexisting ions, and reflects the interplay between valence, concentration, and self-diffusion coefficients of ions involved.

Faster anion diffusion can be hindered by imposing binary diffusion conditions, forcing anions to diffuse at the same rate as cations. Therefore, effective barriers to mitigate both cation and anion transport should have not only low permeability but also a low surface charge and low excess salts to minimize the preexisting ionic concentration.

## Supplementary Material

pgae366_Supplementary_Data

## Data Availability

Excel files containing the raw data will be made available in the Dryad repository with DOI: 10.5061/dryad.x69p8czrt upon publication. All the data are represented in the figures presented in the main manuscript and supplementary figures.
