# Supplementary material for: Understanding the limits of binary diffusion for enhanced clay barrier design
Source: PNAS Nexus. 2024 Aug 23;3(9):pgae366. doi: 10.1093/pnasnexus/pgae366 (PMC11388101; doi:10.1093/pnasnexus/pgae366)
Supplement: pgae366_Supplementary_Data [file pgae366_supplementary_data.pdf]

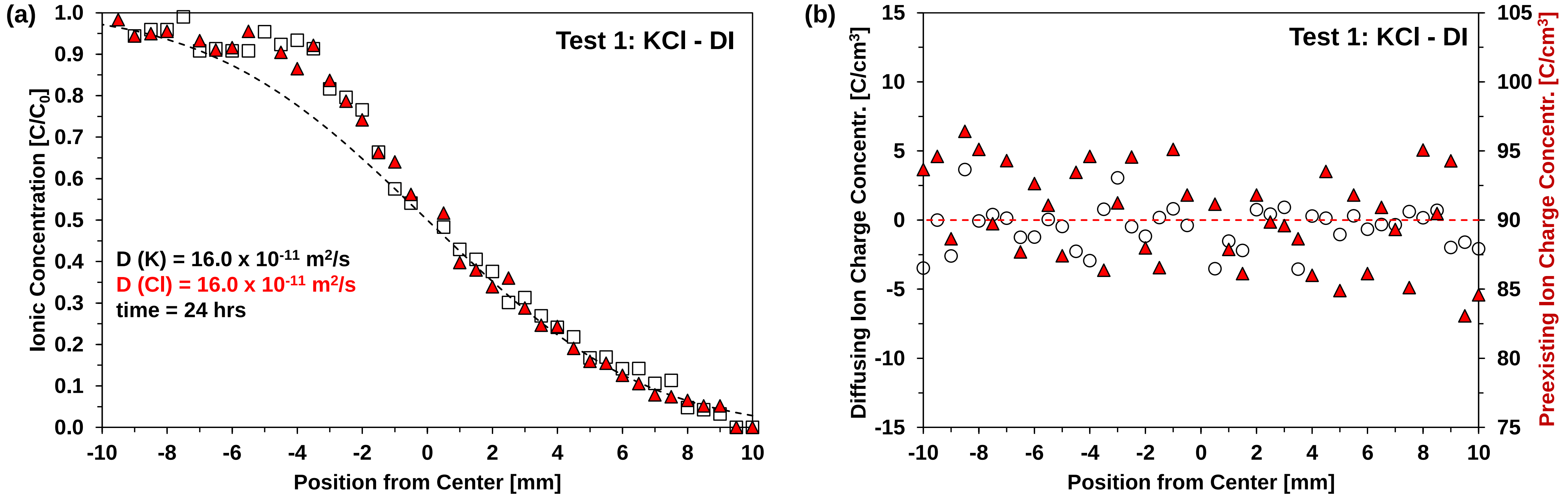

Fig S1. Test 1 (a) Concentration profile of diffusing ions (b) Charge distribution of diffusing and preexisting ions

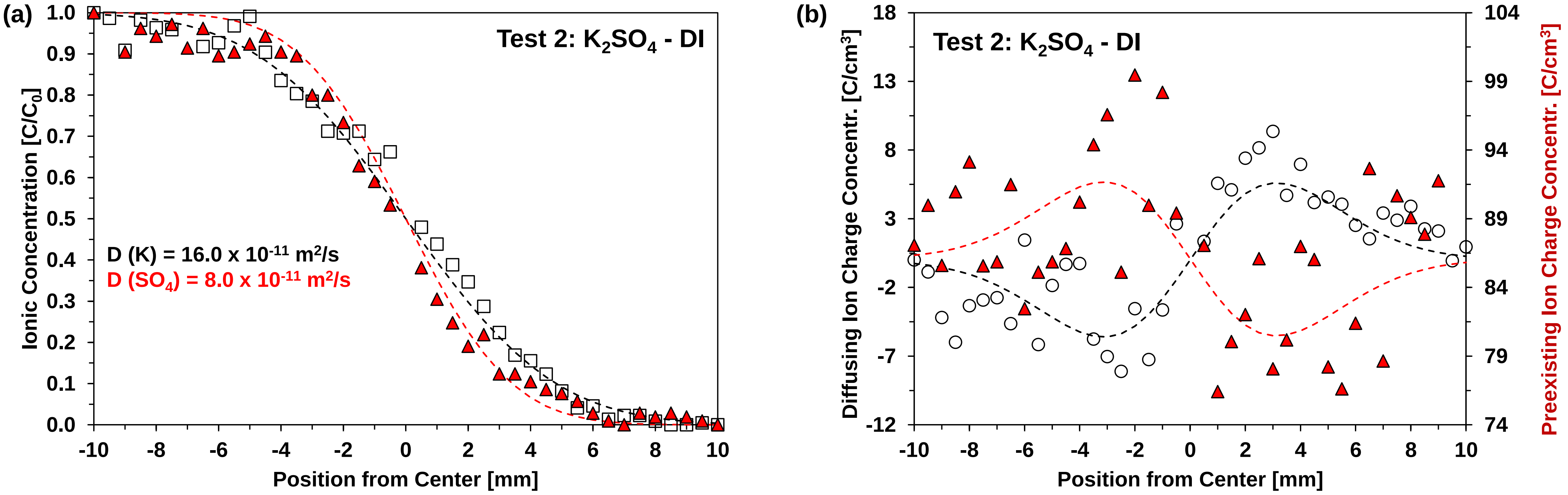

Fig S2. Test 2 (a) Concentration profile of diffusing ions (b) Charge distribution of diffusing and preexisting ions

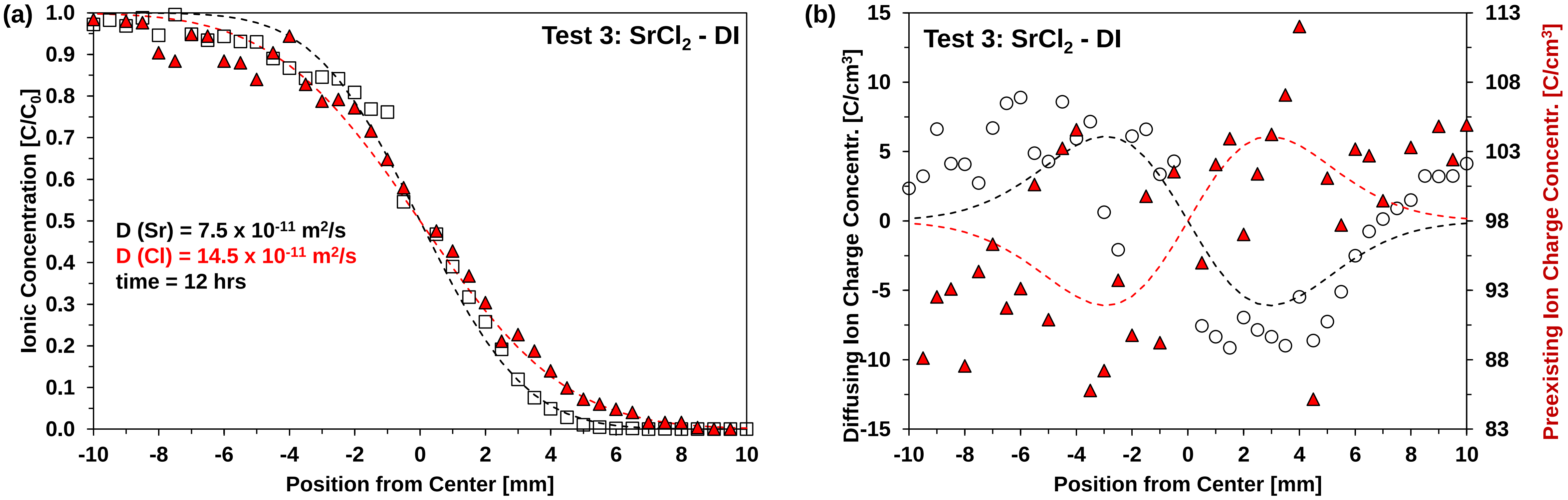

Fig S3. Test 3 (a) Concentration profile of diffusing ions (b) Charge distribution of diffusing and preexisting ions

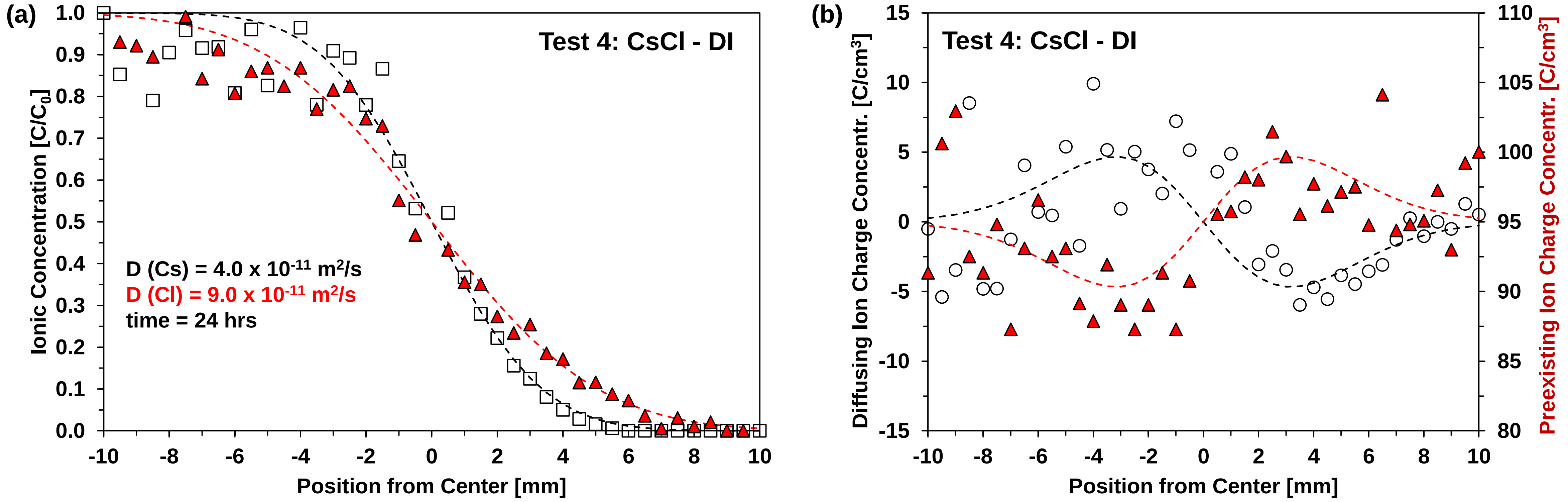

Fig S4. Test 4 (a) Concentration profile of diffusing ions (b) Charge distribution of diffusing and preexisting ions

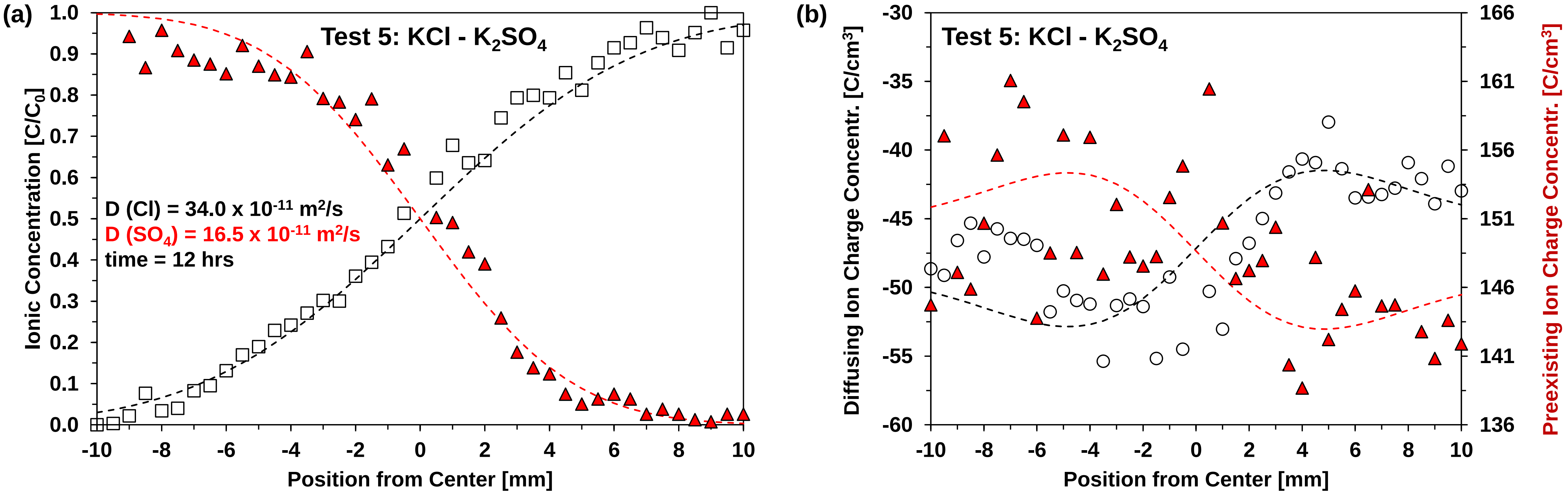

Fig S5. Test 5 (a) Concentration profile of diffusing ions (b) Charge distribution of diffusing and preexisting ions

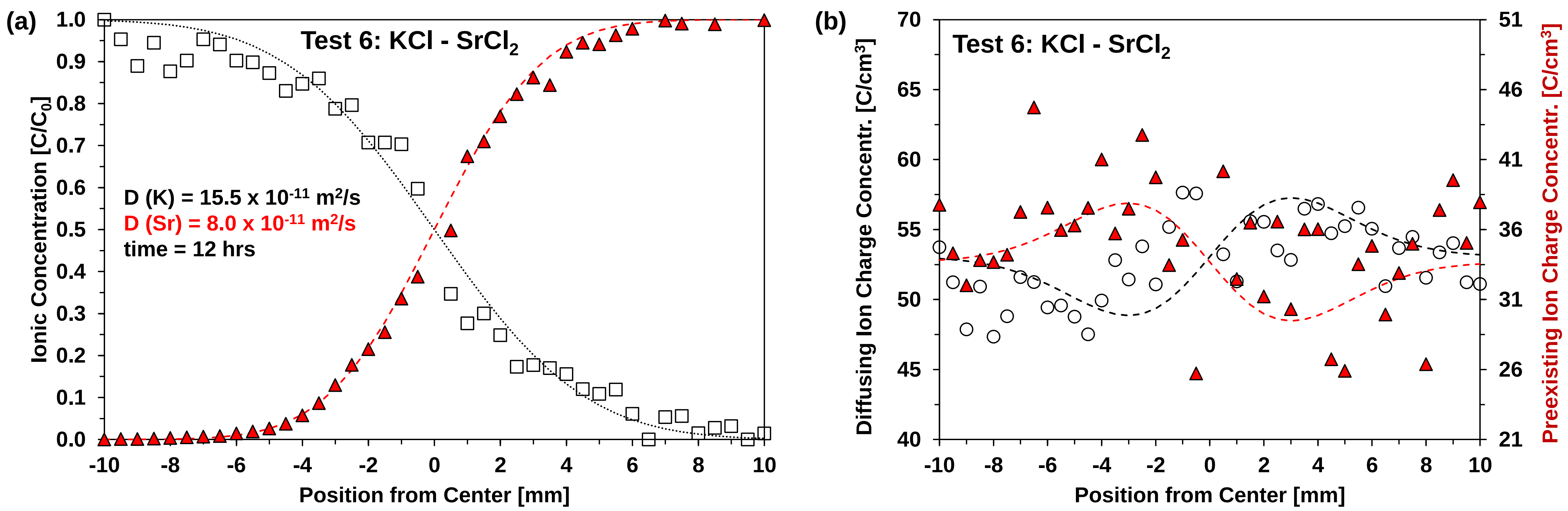

Fig S6. Test 6 (a) Concentration profile of diffusing ions (b) Charge distribution of diffusing and preexisting ions

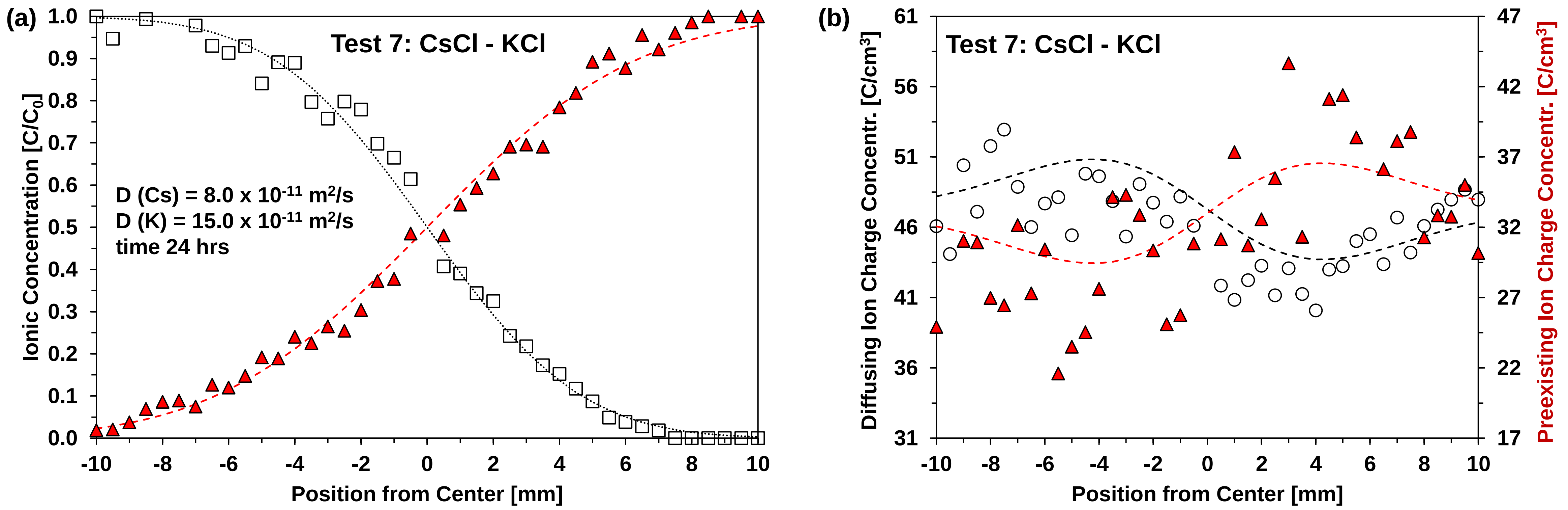

Fig S7. Test 7 (a) Concentration profile of diffusing ions (b) Charge distribution of diffusing and preexisting ions

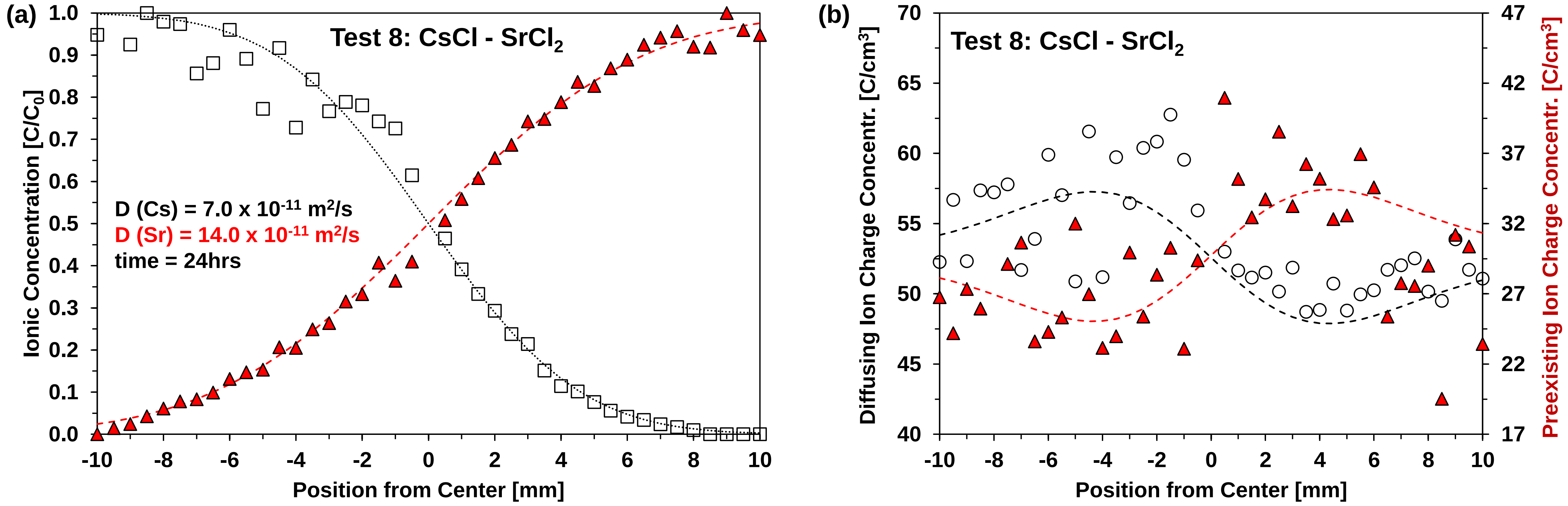

Fig S8. Test 8 (a) Concentration profile of diffusing ions (b) Charge distribution of diffusing and preexisting ions

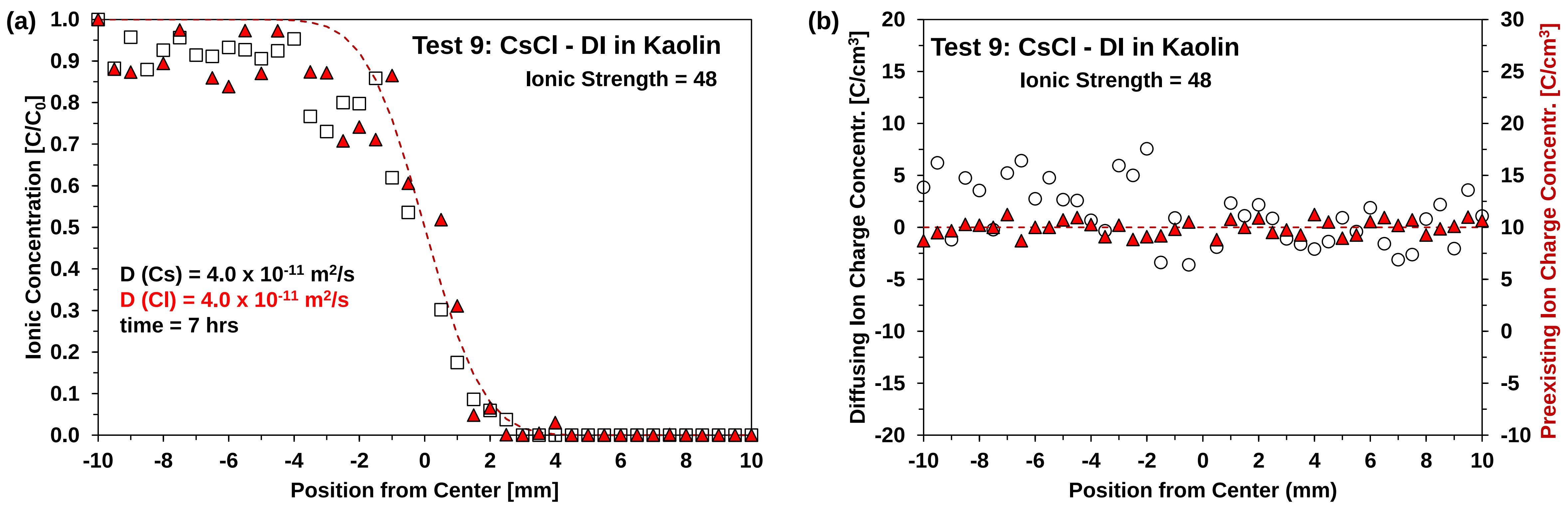

Fig S9. Test 9 (a) Concentration profile of diffusing ions (b) Charge distribution of diffusing and preexisting ions

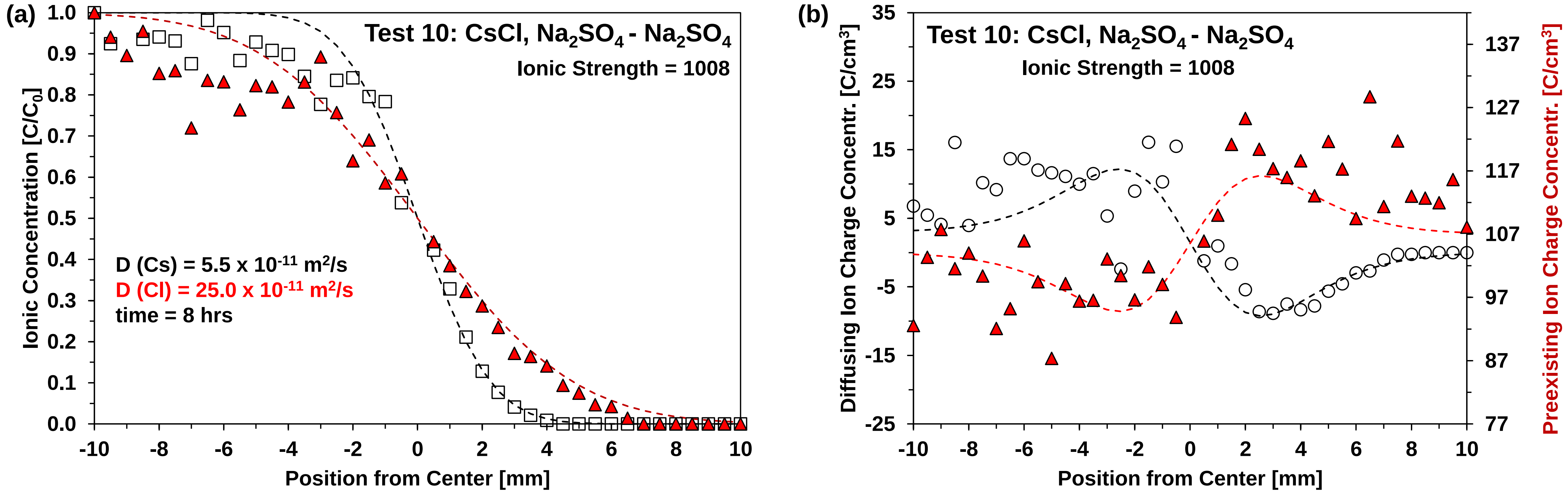

Fig S10. Test 10 (a) Concentration profile of diffusing ions (b) Charge distribution of diffusing and preexisting ions

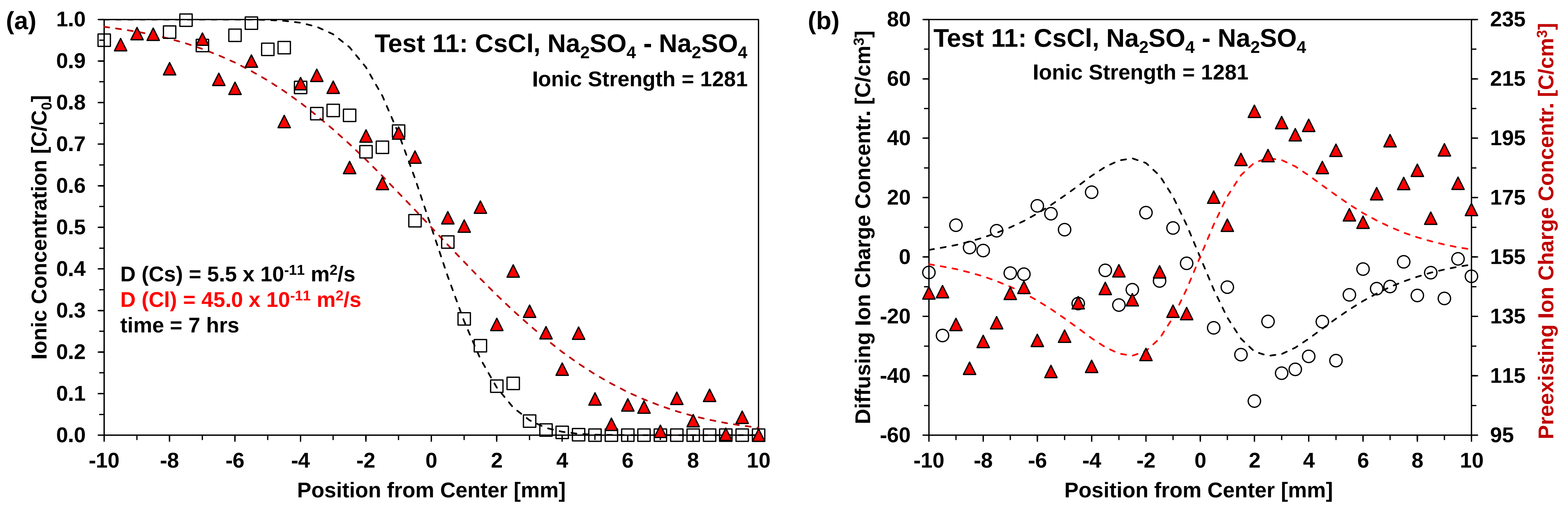

Fig S11. Test 11 (a) Concentration profile of diffusing ions (b) Charge distribution of diffusing and preexisting ions

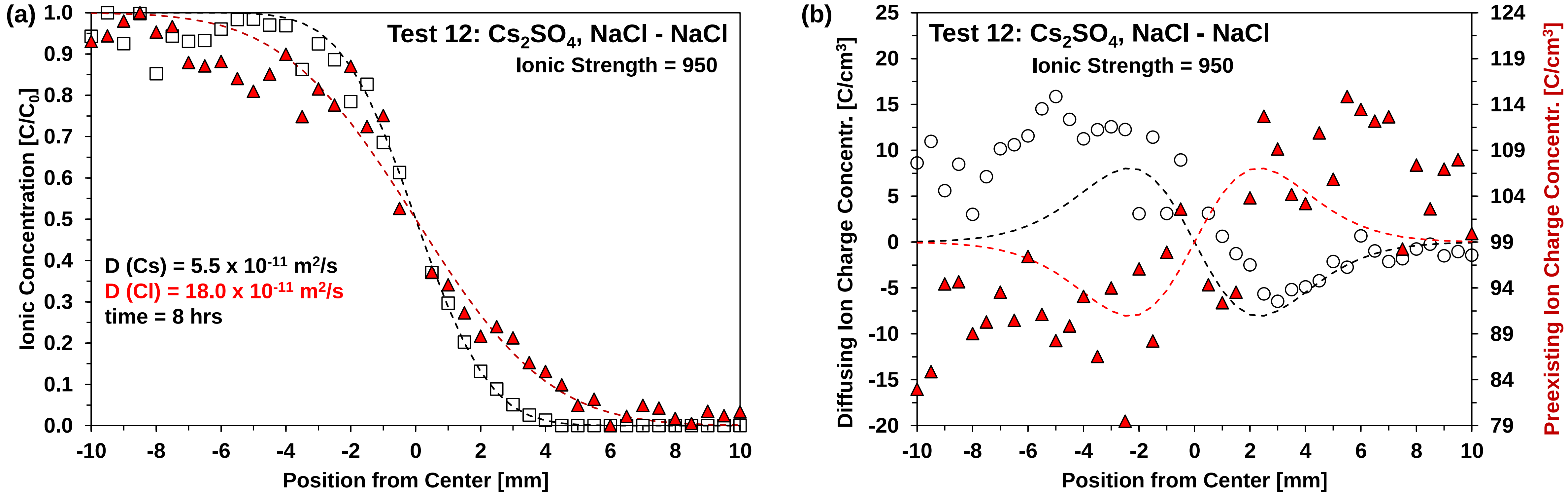

Fig S12. Test 12 (a) Concentration profile of diffusing ions (b) Charge distribution of diffusing and preexisting ions

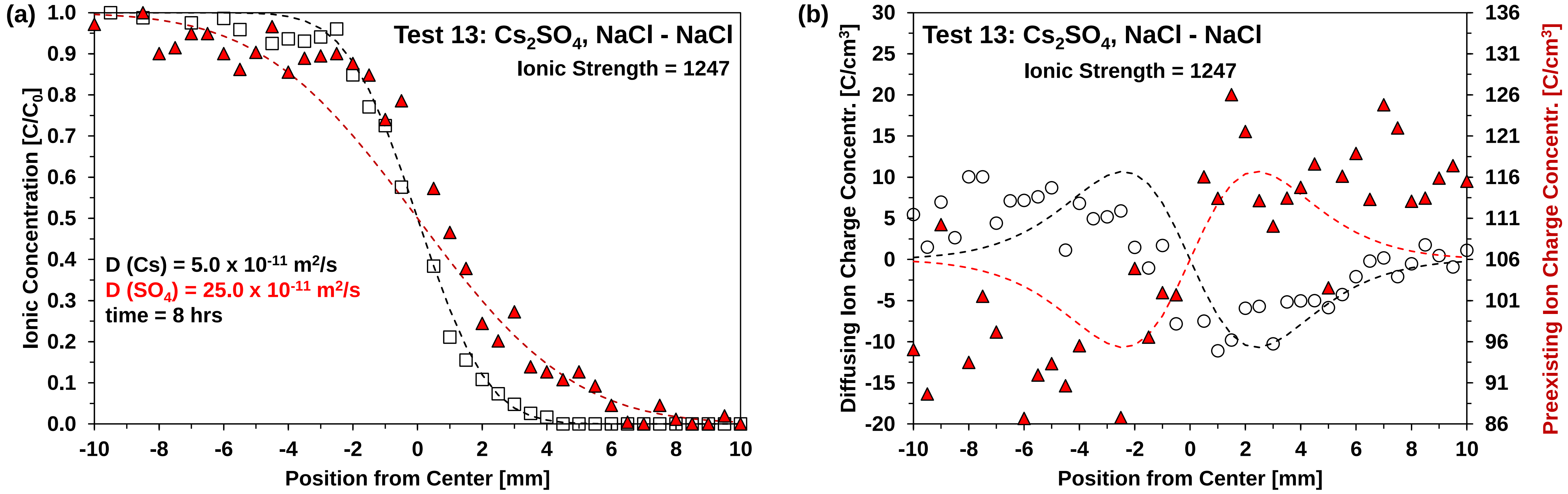

Fig S13. Test 13 (a) Concentration profile of diffusing ions (b) Charge distribution of diffusing and preexisting ions

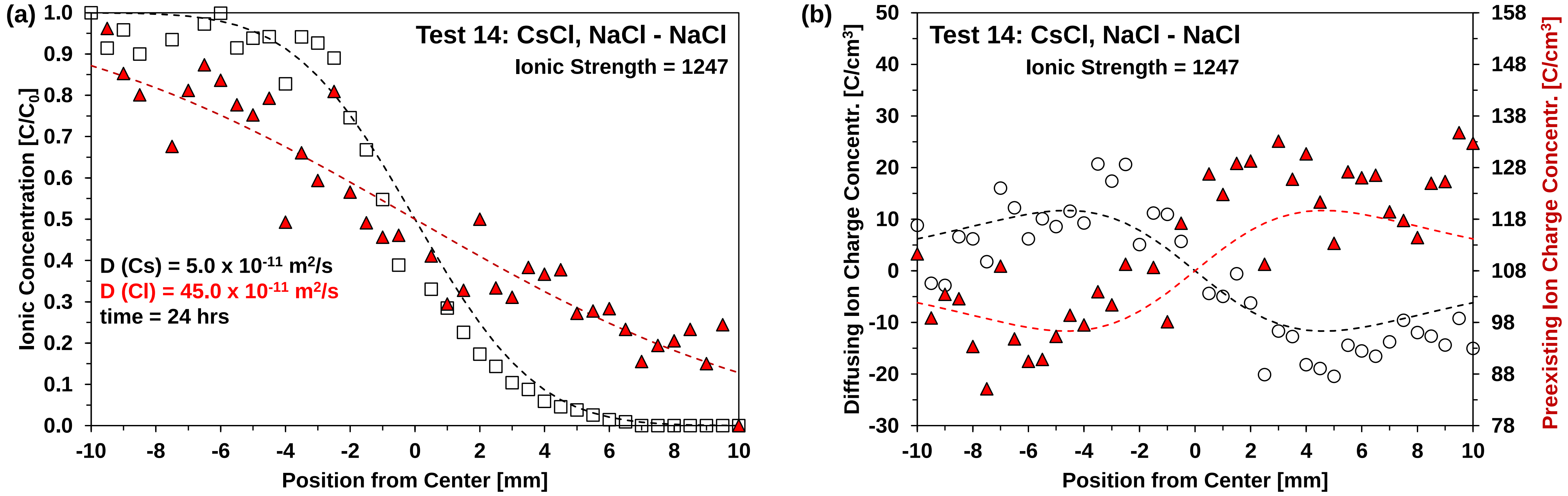

Fig S14. Test 14 (a) Concentration profile of diffusing ions (b) Charge distribution of diffusing and preexisting ions
